# L Chromosome Behaviour and Chromosomal Imprinting in *Sciara Coprophila*

**DOI:** 10.3390/genes9090440

**Published:** 2018-09-03

**Authors:** Prim B. Singh, Stepan N. Belyakin

**Affiliations:** 1 Nazarbayev University School of Medicine, 5/1 Kerei, Zhanibek Khandar Street, Astana Z05K4F4, Kazakhstan; 2 Epigenetics Laboratory, Department of Natural Sciences, Novosibirsk State University, Pirogov str. 2, Novosibirsk 630090, Russia; belyakin@mcb.nsc.ru; 3 Genomics laboratory, Institute of Molecular and Cellular Biology SB RAS, Lavrentyev ave, 8/2, Novosibirsk 630090, Russia

**Keywords:** supernumerary chromosomes, heterochromatin, parent-of-origin effects, paternal X chromosome, maternal X chromosome, controlling element

## Abstract

The retention of supernumerary chromosomes in the germ-line of *Sciara coprophila* is part of a highly-intricate pattern of chromosome behaviours that have fascinated cytogeneticists for over 80 years. Germ-line limited (termed L or “limited”) chromosomes are cytologically heterochromatic and late-replicating, with more recent studies confirming they possess epigenetic hallmarks characteristic of constitutive heterochromatin. Little is known about their genetic constitution although they have been found to undergo cycles of condensation and de-condensation at different stages of development. Unlike most supernumeraries, the L chromosomes in *S. coprophila* are thought to be indispensable, although in two closely related species *Sciara ocellaris* and *Sciara reynoldsi* the L chromosomes, have been lost during evolution. Here, we review what we know about L chromosomes in *Sciara coprophila*. We end by discussing how study of the L chromosome condensation cycle has provided insight into the site and timing of both the erasure of parental “imprints” and also the placement of a putative “imprint” that might be carried by the sperm into the egg.

## 1. Introduction

*Sciara coprophila* possesses a complicated, sometimes bizarre, pattern of chromosomal behaviours that involve both the regular chromosomes and the supernumerary germ-line limited or L chromosomes [1,2,3]. The cycles of both kinds of chromosome are intimately associated. Because of this, L chromosome behaviour is best described as part of the whole. Accordingly, we begin with the newly-fertilized egg in which the well-known X-X’ device in mothers has conditioned the ooplasm to determine the sex of the embryo [4]. Looking at the chromosomal complements provided by each parent, the female pro-nucleus provides 5 chromosomes (3 autosomes, 1 X or X’ chromosome and 1 L chromosome); sperm that forms the male pro-nucleus delivers 6–7 chromosomes (3 autosomes, 2 identical X chromosomes and 1–2 L chromosomes). After syngamy, the zygotic nucleus contains 11–12 chromosomes.

### 1.1. L Chromosome Elimination in the Soma

**Figure 1:**
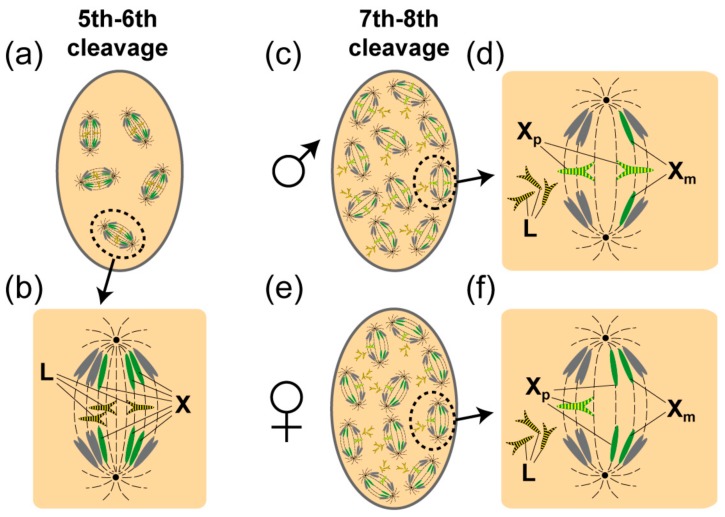
Elimination of L and paternal X chromosomes (Xp) in the embryonic soma. (**a**) Several mitoses are depicted in an embryo at the 5–6th embryonic cleavage where the L chromosomes are in the process of being eliminated. (**b**) One is magnified and shows that that the L chromosomes are left behind at the equatorial plate and fail to separate. The arms of the L chromosomes retain high levels of phosphorylation of histone H3 serine 10 (H3S10P) (shown as yellow stripes). (**c**) In males at the 7–8th cleavage two Xps are eliminated. (**d**) As with the eliminated L chromosomes, the arms of the eliminated Xps fail to separate and likewise retain high levels of H3S10P (shown as yellow stripes). The Xps remain at the equatorial plate and are eliminated giving a somatic male (XO) chromosome constitution. In (**d**) phosphorylated L chromosomes that have already been eliminated are shown. (**e**) In females, a single Xp is eliminated at the 7th–8th cleavage division. (**f**) The single Xp remains at the equatorial plate and retains high levels of H3S10P (shown as yellow stripes). Note that by contrast to the eliminated chromosomes the chromosomes that move away to the poles have lost H3S10P. Eliminations normally take place at the periphery of the multinucleate cell but because the diagram is in 2 dimensions mitoses are seen to fill the cell. The L chromosomes are black, the autosomes are grey and the Xps are green. Phosphorylation is depicted as yellow stripes.

Cleavage divisions take place within a multinucleate syncytium and it is around the fifth to sixth division that the L chromosomes are eliminated from somatic nuclei of both males and females (Figure 1) [5]. Elimination results from a failure of L chromosomes to move pole-ward like the ordinary chromosomes. They are arrested in their anaphase separation and left behind at the equatorial plate to be found only later, discarded as amorphous clumps of chromatin at the periphery of the multinucleate cell. By contrast, L chromosomes are retained in the 2–4 primordial germ cells found at the posterior of the coenocyte after the fifth division. In this way, L chromosomes become limited to the germ line.

After the somatic elimination of the L chromosomes there are nine chromosomes (6 autosomes and 3 X chromosomes) in somatic cells; the germ line contains 11–12 since the L chromosomes are retained. Strikingly, the eliminations in the soma do not end there. Another elimination event is programmed to take place soon after, which is regulated by the X-X’ mechanism that determines the sex of the embryo. At the seventh or eighth cleavage division, there is an elimination of X chromosomes, which are exclusively paternal in origin [1]. The somatic eliminations are complete at around 4–6 h post oviposition (see Table 16 in [6]). Thereafter the number of chromosomes is reduced to seven in the male soma (XO), because two paternal X chromosomes (Xps) are lost along with all the L chromosomes and to eight chromosomes in the female soma (XX or X’X), where one Xps is lost along with all L chromosomes (Figure 1d,e). 

After the eliminations in the soma, germ cells undergo another division that is followed by a long resting stage during which the germ cells migrate to the site of the presumptive gonad. Soon after reaching the gonad another round of eliminations takes place. 

### 1.2. L Chromosome Elimination in the Germ-Line

**Figure 2:**
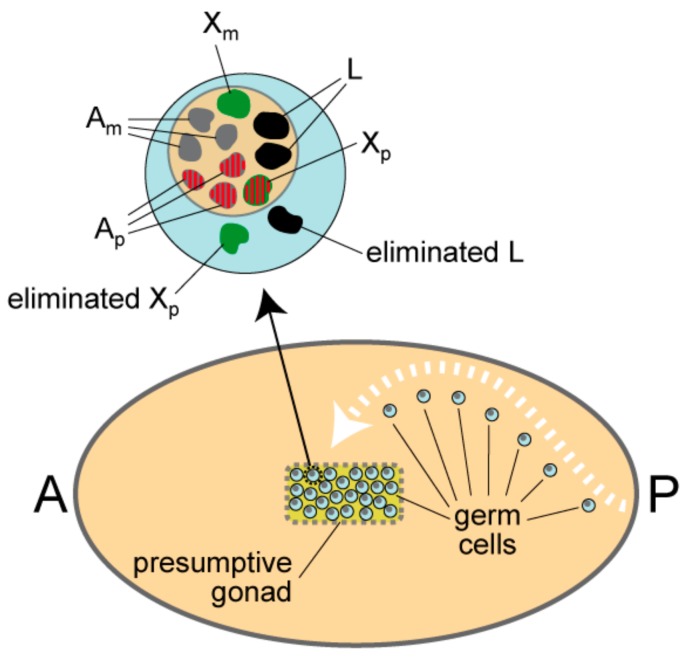
Elimination of L and Xp chromosomes in resting germ cells of the presumptive gonad. After rounds of cell division at the posterior of the embryo, germ cells enter a quiescent state and migrate to the presumptive gonad. Around 30 arrive in the gonad. In the gonad of both sexes, the chromosomes in the resting germ cells form distinct entities called pro-chromosomes, as shown in the magnified germ cell above the embryo. All but two L chromosomes are eliminated and one Xp is eliminated. The eliminated chromosomes lie in the cytoplasm. In the nucleus, the three paternal autosomes and the retained Xp are acetylated (acetylation is shown as red stripes); these four paternal chromosomes are also cytologically more diffuse than all the other chromosomes. Notably, the eliminated Xp is under-acetylated, which is also the case for all the other chromosomes. The L chromosomes are black, the autosomes are grey and the X chromosomes are green. Acetylation is shown as red stripes. Am are the maternal autosomes and Ap are the paternal autosomes. Xm is the maternal X chromosome and Xp is the paternal X chromosome. A is anterior and P is posterior.

Between 24–48 h post-oviposition the 30 or so resting germ cells are in the process of migrating to the site of the presumptive gonad [6]. Each germ cell possesses three X chromosomes (two paternal and one maternal), the autosomes and all of the L chromosomes. Just prior to or concurrently with elimination, the regular chromosomes in both sexes form fairly discrete bodies calledpro-chromosomes [7], which are differentiated. The four paternal chromosomes are more diffuse and lightly-staining while the maternal homologues are more condensed and darkly-stained [6]. The fifth paternal chromosome, which is the extra X chromosome, remains condensed and it is this chromosome that is eliminated at 60–72 h post-oviposition from the germ cells in both sexes. Along with the paternal X chromosome all but two L chromosomes are eliminated [6]. Notably, the L chromosomes, which are typically heterochromatic and darkly-stained, become diffuse and lightly-strained at the same time as the paternal chromosome set [6]. After the eliminations two L chromosomes and eight regular chromosomes remain (Figure 2).

The mechanism for elimination in germ cells is very different to that which takes place in soma of the early embryo (Figure 1). The germ cells have an intact nuclear membrane and elimination involves passage of whole chromosomes through the nuclear membrane into the cytoplasm where they become very smooth and round in contour and remain darkly-stained until they disappear. How passage through the membrane is achieved is not known, although the close apposition of the Xp to be eliminated to the nuclear membrane has led to the suggestion that membrane-spanning proteins, which interact with silenced chromatin—such as the lamin B receptor [8]—might be involved [9]. Elimination of the X chromosome may precede or follow L chromosome elimination in germ cells, unlike the strict order of elimination for the two kinds of chromosomes in the soma [6].

### 1.3. L Chromosome Behaviour During Male Meiosis

Meiosis is initiated during pupation some 23 days after oviposition [6]. In females, it is orthodox. The L chromosomes also pair and undergo the typical reduction divisions. In stark contrast, in males, both meiotic divisions are unequal, the first to a striking degree [1,2,3,10]. The first meiotic division is monocentric and brings about the selective elimination of the regular paternal homologues. 

**Figure 3:**
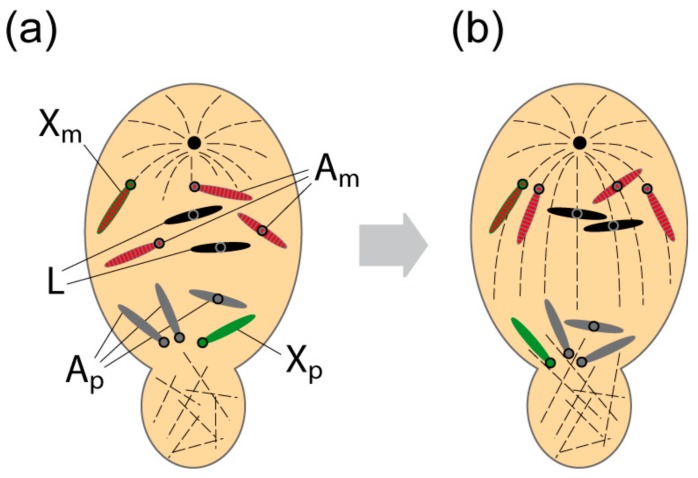
Retention of L and regular maternal homologues during first meiotic division in primary spermatocytes. (**a**) At meiotic prophase, the L chromosomes and regular maternal chromosomes display a similar yet separate localisation to the paternal homologues within the nucleus. A monopolar spindle is formed and non-spindle microtubules are generated in the cytoplasmic bud region. The maternal chromosomes are acetylated (acetylation is shown as red stripes). (**b**) The chromosomes move directly to an anaphase like stage where the L chromosomes and regular maternal chromosomes move towards the single pole while paternal homologues segregate into the bud. The maternal chromosomes are acetylated. Circles represent the positions of the centromeres. The L chromosomes are black, the autosomes are grey and the X chromosomes are green. Acetylation is shown as red stripes.

Accordingly, the four maternally-derived regular chromosomes move to the single pole and notably, along with them, move all L chromosomes of which there are usually two in number (Figure 3). Unlike the ordinary chromosomes, the parental origin of the L chromosomes is immaterial: The L chromosomes retained in the germ-line can be derived from either parent [6,11]. Thus, with regard to the L chromosomes, the first meiotic division in males is not a reduction division and they are included in unreduced number in the secondary spermatocyte and thereafter in the sperm [6]. The reason for the germ-line eliminations described above (Figure 2) becomes plain; in their absence, the number of L chromosomes would increase over successive generations. 

**Figure 4:**
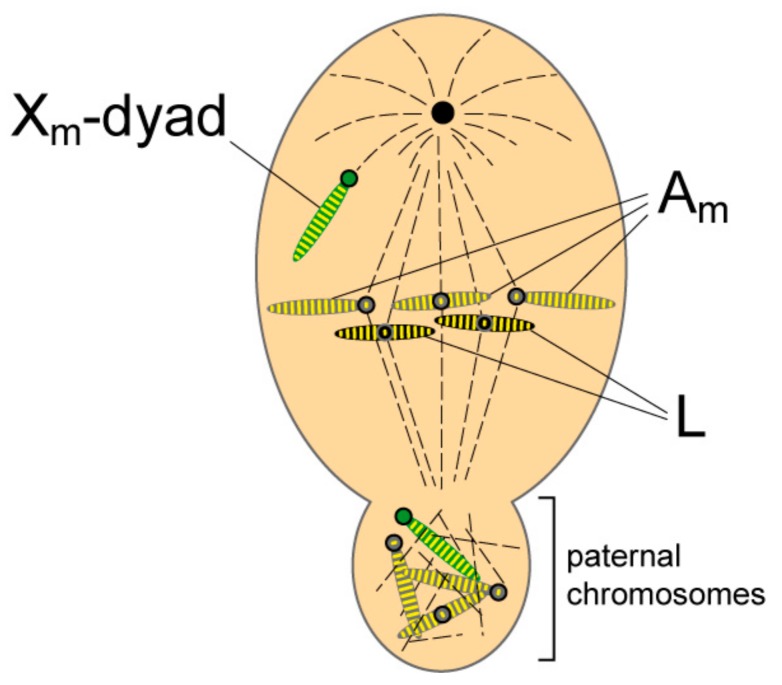
Lack of phosphorylation of the X centromere region at meiotic metaphase II. The Xm-dyad is seen precociously at the monopole. The arms of Xm retain histone histone 3 (H3) phosphorylation (given as yellow stripes), while the centromere region (the ribosomal DNA (rDNA)-chromosomal tip) is devoid of phosphorylation (no stripe in the X centromere). The regular maternal homologues and the L chromosomes lie on the metaphase plate and exhibit phosphorylation of histone H3 both along the arms and the centromere regions. The regular paternal chromosomes found in the bud are also phosphorylated except for the centromere region of Xp. Circles indicate centromere position on the chromosomes. The L chromosomes are black, the autosomes are grey and the X chromosomes are green. Phosphorylation is shown as yellow stripes.

The second meiotic division is essentially orthodox except that the X-chromosome undergoes non-disjunction and is found precociously at the monopole (Figure 4). The X-dyad then passes into the sperm nucleus while the chromosome group devoid of X chromosomes is extruded and degenerates. At the end of the asymmetric meiosis the spermatocyte gives rise to one sperm cell and a bud containing the eliminated chromosomes. 

Studies on the sciarid species, *Trichosia pubescens*, have shown that in spermatocytes, L chromosomes become de-condensed and indistinguishable from ordinary chromosomes during the interphase between the meiotic divisions [12]. Based on this observation it was suggested that the de-condensation represents gene activity likely required for proper spermatogenesis, a notion supported by the unpublished results of Crouse (cited in [6]) who observed that spermatocytes having no L chromosomes were smaller, develop more slowly and do not undergo typical (albeit unequal) meiosis. However, this conclusion must be tempered by the observation that both *Sciara ocellaris* and *Sciara reynoldsi* do not possess L chromosomes [6,11]. It has been argued that genes on L chromosomes required for spermatogenesis may have been transferred onto the autosomes in these closely-related species during evolution rendering the former dispensable [6].

## 2. The Epigenotype of L Chromosomes

A series of detailed studies by Goday and co-workers has led to a description of the localisation of epigenetic modifications (histone and DNA) and the non-histone chromosomal protein, heterochromatin protein 1 (HP1), on ordinary and L chromosomes [9,13,14,15,16]. The epigenetic modifications associated with L chromosomes are given in Table 1. L chromosomes possess modifications that are characteristic of constitutive heterochromatin (for reviews see [17,18]). There is a lack of acetylation on histones histone 3 (H3) and H4, a modification associated with gene activity, while there is an over-representation of repressive histone marks such as methylation of lysines 9 on H3 and 20 on H4 as well as an enrichment of 5-methyl-cytosine in chromosomal DNA. Also enriched are two HP1 homologues, ScoHET1 and ScoHET2; HP1 is a hallmark of heterochromatin [19].

Study of histone phosphorylation has shown an association with L chromosomes eliminated in the soma. Localisation of phosphorylation on serine 10 of histone H3 (H3S10P) on L chromosomes undergoing elimination revealed that the levels remain high along the chromatid arms ([16]; Table 1). By comparison, the levels on the ordinary chromosomes have fallen [16]. The retention of H3S10P levels are also observed along the arms of the Xp chromosomes during their elimination from the soma [16]. This has led to the suggestion that inhibition of de-phosphorylation of the chromatid arms is part of the mechanism that orchestrates the programmed elimination of both kinds of chromosome from the soma during the embryonic cleavages [16]. 

Histone phosphorylation of L chromosomes in spermatocytes during the meiotic divisions is almost identical to that found for the regular maternal chromosomes ([15]; Table 1), indicating that similar mechanisms might operate to retain both kinds of chromosomes in the face of the selective elimination of regular paternal chromosomes at meiosis I. However, the mechanisms involved cannot be identical. This is because the regular chromosomes retained in spermatocytes during the first meiotic division are exclusively maternal in origin, while the L chromosomes that move en bloc to the same monopole can be derived from either parent [6,11]. How the paternal L chromosomes are stopped from moving backward along with the four ordinary paternal homologues is not known.

The parent-of-origin behaviours of the regular chromosomes in *Sciara* have been of great interest for many decades (for reviews see [1,2,3]). Notably it has been suggested that the study of L chromosome behaviour may provide insight into the mechanisms of so-called chromosomal imprinting [20] of the regular chromosomes in *Sciara*.

## 3. L Chromosomes and Chromosomal Imprinting of the Regular Chromosomes

Chromosomal imprinting was a term coined by Helen Crouse [20] to describe the reversible identification of homologues that exhibit the parent-of-origin-specific behaviour seen in spermatocytes, where the four regular paternal homologues are selectively eliminated during meiosis I (Figure 3). Imprinting is reversible because the maternal homologues that are retained and enter the sperm become the paternal chromosomes that behave in precisely the opposite manner at meiosis I in sons. The selective elimination of Xp chromosomes [1] in soma and germ-line provides another example of chromosome imprinting. Notably, the X-X’ device operating in the mother determines the sex of the soma by ensuring the correct number of Xp chromosomes are eliminated [21]. The mechanism of elimination itself requires a chromosomal segment embedded in the constitutive heterochromatin adjacent to the X centromere called the controlling element (CE; [20]). 

Strikingly, while L chromosomes also undergo elimination in both soma and germ-line (Figure 1 and Figure 2), they are not subject to chromosomal imprinting, which led Crouse et al. [11] to conclude that: “…in Sciara there are two independent systems of chromosome identification: one which distinguishes supernumerary from regular; the other which identifies regular as paternal or maternal.”

Based on the condensation cycle of both L and regular chromosomes Crouse et al. [6,11] attempted to identify specific germ-line stages where the identification (imprinting) process might be regulated. It is worth revisiting those analyses.

### 3.1. Erasure of Parental Imprints and Selective Retention of L Chromosomes and Regular Maternal Chromosomes at the First Spermatocyte Division

The de-condensation of the L-chromosomes, along with the regular paternal chromosomes, in or around the time of the germ line eliminations (Figure 2) was suggested to represent the time at which the parent-of-origin imprint on the regular chromosomes is erased [6]. Erasure ensures no difference between the regular chromosomes thus enabling meiosis to proceed in an orthodox manner in females. This timing is supported by studies on histone acetylation that have shown the de-condensed paternal chromosomes (except for the Xp to be eliminated) are initially hyper-acetylated [9] and thereafter, upon entry into the gonal mitotic divisions, both parental sets become equally acetylated [9]. Notably, the de-condensed L chromosomes remain under-acetylated (Table 1). That the L chromosomes are out-of-step with the regular chromosomes is most likely because L chromosomes are not imprinted. 

Given that erasure might take place in resting spermatocytes, how are the chromosomes identified later at the first spermatocyte division? A mechanism for identification must exist because paternal homologues are selectively eliminated while the maternal homologues and L chromosomes are retained. How this might be achieved comes from the observation that elimination does not require homologue pairing and metaphase alignment–instead the chromosomes proceed directly to an anaphase-like stage [2,3]. There is an intrinsic separation of the chromosomes that follow different fates. This is so even in early germ cells where there is non-random clustering of the ordinary maternal homologues and L chromosomes in a nuclear compartment distinct to that occupied by the paternal homologues [9,22,23]. It remains to be proven but it may be that this compartmentalisation presages and of itself determines the later selective elimination of the regular paternal homologues. 

In primary spermatocytes, there is also a reversal in the acetylation status of the ordinary chromosomes where the maternal homologues retain their high levels of acetylation while the paternal homologues become under-acetylated [9]. As explained, loss of acetylation is a feature of the Xp eliminated in the germ-line (Figure 2), indicating that the loss of acetylation is a characteristic of the parent-of-origin specific elimination of the ordinary paternal homologues. Interestingly, the non-imprinted L chromosomes are under-acetylated and thus out-of-step with the imprinted homologues that accompany them to the monopole [9]. 

In secondary spermatocytes, the L chromosomes exhibit a high degree of H3 histone phosphorylation (Table 1; [15]). This is also true for the maternal homologues except for the centromeric ribosomal DNA (rDNA) region of the X-dyad, which includes the CE, where there is a clear deficiency in H3 phosphorylation (Figure 4; [15]).

### 3.2. L Chromosomes and Imprinting of the Controlling Element

The CE regulates two different events. First, the meiotic non-disjunction of the precocious maternal X-dyad in secondary spermatocytes by centromere inactivation (Figure 4) and, second, the elimination of the now paternal X chromosomes in the embryo and germ lines (Figure 1 and Figure 2). X-autosome translocations have shown that the CE represents the site of imprinting that enables the Xp to be distinguished from the Xm in soma and germ cells. The act of imprinting itself requires that the imprint is placed on a chromosome at the time when the parental genomes are separate, which can be in the respective germ-lines or in the brief period when the parental genomes lie separately within the pro-nuclei of the newly-fertilized egg. It has been suggested that if the maternal X chromosomes in the sperm carry a paternal imprint into the egg it is likely that the imprinting event takes place at the end of anaphase in meiosis II, when L chromosomes reach their greatest condensation–and therefore most likely to be resistant to imprinting–and the regulars are de-condensed and diffuse [see discussion in 11]. Alternatively, imprinting may take place in the ooplasm. This has been suggested on two grounds, first, because the X-X’ device in the mother conditions the ooplasm to eliminate the appropriate number of Xp chromosomes [4] and, second, by analogy with the situation in Coccids where imprinting is regulated by the mother with no contribution from the father [24]. 

Recently, a molecular model of how a CE can cause the elimination of an Xp during the early cleavage divisions has been posited (see Figure 2B in [25]). According to the model, the CE encodes a non-coding RNA (ncRNA) that acts upon the Xp’s chromosome arms, leading to their failure to separate at the anaphase of the 7th to 8th cleavage division and subsequent elimination. Research in *Sciara coprophila* is entering an exciting phase for it may now be possible with the advent of molecular techniques to dissect the imprinting process and, in addition, to elucidate the mechanism(s) by which the L chromosomes avoid it.

## Figures and Tables

**Table 1:** Epigenetic modifications associated with the supernumerary L chromosomes at different stages of development. L chromosomes have epigenetic profile characteristic of constitutive heterochromatin. Apart from the first somatic mitosis, the L chromosomes are de-acetylated. Where investigated, L chromosomes also share the repressive epigenetic modifications and non-histone heterochromatin protein 1 (HP1) chromosomal proteins. Analyses of phosphorylation show that during the somatic eliminations the arms of the L chromosomes retain phosphorylation with H3S10P. During meiosis L chromosomes show a complex pattern of phosphorylation (see text for details). Data for acetylation is taken from [9]. The distribution of repressive epigenetic modifications and HP1 proteins was taken from Figures 6 and 7 of [13] in “pre-meiotic” and “young germ nuclei” that are likely to be in or around larval mitosis I. For description of phosphorylation during the meiotic divisions, the data was taken from [15]. For phosphorylation of L chromosomes in the embryonic soma the data was taken from [16].

Stage	Epigenetic Modifications
First somatic division	L chromosomes are positive for H4K8Ac [9]
Syncytial embryo before elimination	L chromosomes are positive for H3S10P at pro-metaphase [16]. Proceeding to metaphase they become positive for H3S28P [16]. The centromeres are specifically stained with H3T3P antibodies at metaphase [16].
Syncytial embryo during elimination	L chromosome arms are stained with H3S10P at metaphase/anaphase [16]. By contrast, the regulars have lost their H3S10P staining at anaphase.
Resting stage germ cells before elimination of Xp	L chromosomes are negative for H4K8Ac, H4K12Ac and PanH3Ac [9].
Resting stage germ cells after elimination of Xp	L chromosomes are negative for H4K8Ac, H4K12Ac and PanH3Ac [9].
Germ cells undergoing larval mitosis I (9th day after oviposition)	L chromosomes are negative for H4K8Ac [9]. They are positive for H3K9me2, H3K9me3, H4K20me3, 5MeC and the two HP1-like proteins ScoHET1 and ScoHET2 [13].
Germ cells at end of mitosis III in third instar larvae (12th day after oviposition)	L chromosomes are negative for H4K8Ac [9].
Prophase of Meiosis I	L chromosomes are negative for H3S10P, H3S28P and H3T3P but positive for H3T11P [15]
Meiosis I	L chromosomes are negative for H3S10P, H3S28P and H3T3P but positive for H3T11P [15]. They are also negative for H4K8Ac [9].
Meiosis II	L chromosomes are positive for H3S10P, H3S28P and H3T3P and H3T11P [15]. They are also positive for ScoHET1 [13].
